# Systematic Analysis of *Dof* Gene Family in *Prunus persica* Unveils Candidate Regulators for Enhancing Cold Tolerance

**DOI:** 10.3390/ijms26157509

**Published:** 2025-08-04

**Authors:** Zheng Chen, Xiaojun Wang, Juan Yan, Zhixiang Cai, Binbin Zhang, Jianlan Xu, Ruijuan Ma, Mingliang Yu, Zhijun Shen

**Affiliations:** Jiangsu Academy of Agricultural Sciences/Jiangsu Key Laboratory for Horticultural Crop Genetic Improvement, Institute of Pomology, 50 Zhongling Street, Nanjing 210014, China

**Keywords:** 5-aminolevulinic acid (ALA), cold stress, *PpDofs*

## Abstract

Late-spring frost events severely damage low-chill peach blossoms, causing significant yield losses. Although 5-aminolevulinic acid (ALA) enhances cold tolerance through the PpC3H37-PpWRKY18 module, the regulatory mechanism of ALA on *PpC3H37* remains to be elucidated. Using yeast one-hybrid screening with the *PpC3H37* promoter as bait, we identified PpDof9 as a key interacting transcription factor. A genome-wide analysis revealed 25 *PpDof* genes in peaches (*Prunus persica*). These genes exhibited variable physicochemical properties, with most proteins predicted as nuclear-localized. Subcellular localization experiments in tobacco revealed that *PpDof9* was localized to the nucleus, consistent with predictions. A synteny analysis indicated nine segmental duplication pairs and tandem duplications on chromosomes 5 and 6, suggesting duplication events drove family expansion. A conserved motif analysis confirmed universal presence of the *Dof* domain (Motif 1). Promoter cis-element screening identified low-temperature responsive (LTR) elements in 12 *PpDofs*, including *PpDof1*, *PpDof8*, *PpDof9*, and *PpDof25*. The quantitative real-time PCR (qRT-PCR) results showed that *PpDof1*, *PpDof8*, *PpDof9*, *PpDof15*, *PpDof16*, and *PpDof25* were significantly upregulated under low-temperature stress, and this upregulation was further enhanced by ALA pretreatment. Our findings demonstrate ALA-mediated modulation of specific *PpDof* TFs in cold response and provide candidates (*PpDof1*, *PpDof9*, *PpDof8*, *PpDof25*) for enhancing floral frost tolerance in peaches.

## 1. Introduction

The peach (*Prunus persica* L.), a diploid stone fruit species (2n = 16) classified within the *Prunus* genus of the *Rosaceae* family, originated in the northwest region of China approximately 4000–5000 years ago based on archeological and molecular evidence [1,2]. Its fruit exhibits vibrant coloration, a distinct aroma, a unique flavor profile, and significant nutritional density, underpinning its broad consumer popularity [3]. Global warming is reducing winter chilling accumulation for peaches, necessitating the development of low-chill cultivars to adapt to rising temperatures [4]. Peach varieties with low chilling requirements exhibit early flowering phenology and heightened susceptibility to low-temperature injury. During natural endodormancy, peach trees demonstrate substantial cold hardiness; the northern germplasm tolerates temperatures of −23 to −26 °C, while the southern germplasm withstands −15 °C temperatures, albeit with occasional trunk damage in northern regions. Post-dormancy, the sensitivity to frost increases markedly; floral buds and open flowers incur damage at −1.7 °C and −1.1 °C, respectively. Low-chill varieties typically flower in early spring. The flowering period of the peach is relatively short and highly sensitive to temperature [5]. Late-spring cold events, typically occurring around March, induce abrupt temperature declines during the anthesis of low-chill peach cultivars. These suboptimal temperatures cause severe floral freezing injury, leading directly to significant annual yield losses in commercial production [6]. With the increasing frequency of late-spring cold events in recent years, enhancing floral frost tolerance in low-chill peaches has become imperative to mitigate economic damage, underscoring the need for targeted research.

Transcription factors play pivotal roles in regulating plant responses to diverse abiotic stresses. Functional studies demonstrate that MbWRKY3 acts as a positive regulator under drought stress [7], while MbMYBC1 responds to both cold and hydropenia signals, enhancing tolerance to low temperature and drought [8]. Furthermore, overexpression of FvMYB114 and FvMYB44 in Arabidopsis thaliana significantly bolstered plant tolerance to combined salt and low-temperature stresses [9,10]. These findings collectively underscore the critical importance of specific transcription factors in mediating plant adaptation to key environmental constraints, including drought, salinity, and cold. Among them, the Dof (DNA binding with one finger) proteins are the specific TFs (transcription factors) in plants, which belong to a subfamily of the zinc lipoprotein family, usually composed of 200 to 400 amino acids, and possess a highly conserved N-terminal Dof domain of 50–52 amino acids, containing a single zinc finger (C2-C2) structure featuring a Cys residue, which recognizes *cis*-regulatory elements harboring the common core sequence 5′-AAAG-3′ [11]. The Dof protein binds specific DNA sequences to regulate gene expression and interacts with protein partners to modulate plant development and abiotic stress responses [12,13]. Dof TFs play evolutionarily conserved roles in cold adaptation mechanisms across diverse plant species. In *Oryza sativa*, the cold-induced Dof1 transcription factor binds to the hypomethylated promoter of *ACT1* (an arabinogalactan protein gene), activating its expression and conferring chilling tolerance during meiosis. This epigenetic regulation mediated by the cold-suppressed DNA methyltransferase MET1b enables stable inheritance of cold-adapted phenotypes for over five generations, providing molecular evidence for Lamarckian ‘acquired inheritance’ in environmental adaptation [14]. The majority of *PheDofs* genes in moso bamboo are involved in response processes to drought, low temperature, and high salinity [15]. However, research on the cold tolerance of the transcription factor Dof in *Prunus persica* has not been reported yet.

5-Aminolevulinic acid (ALA) has garnered increasing research interest for its roles in fruit tree production. Applications include regulating stomatal conductance [16,17], enhancing leaf photosynthesis [18,19], promoting secondary metabolite accumulation [20], improving yield and quality [21], and bolstering resilience to biotic and abiotic stresses [22]. Under abiotic stress, ALA mitigates damage from waterlogging [23], salinity [24], drought [25], and cold [26]. Low temperature critically limits plant productivity. Recent frequent late-spring frost events highlight ALA’s efficacy in protecting low-chill peach blossoms. Yuan et al. (2024) [26] proposed an ALA-regulated PpC3H37-PpWRKY18 module mediating the pistil cold response. Nevertheless, how ALA regulates *PpC3H37* requires further investigation. Using the *PpC3H37* promoter as bait in yeast one-hybrid screening, we identified PpDof9 as an interacting factor. We performed genome-wide identification of *PpDofs*, analyzing their structural characteristics, chromosomal locations, promoter cis-elements, encoded protein physicochemical properties, and expression under cold stress.

## 2. Results

### 2.1. Effects of ALA Treatment on Floral Organs and Physiological and Biochemical Parameters in Peach Blossoms

Our study demonstrates that pretreatment with 50 mg L^−1^ ALA effectively mitigates low-temperature stress damage to peach pistils. Under cold stress, untreated peach flowers exhibited petal dehydration and curling, stamen wilting, anther margin browning or blackening, and pistil browning (style and ovary). In contrast, ALA-pretreated flowers displayed reduced style yellowing or browning and only mild ovary browning under identical stress conditions. These findings indicate that ALA pretreatment partially mitigates low-temperature damage and enhances cold tolerance in peach blossoms (Figure S1A).

An analysis of osmoregulatory substances in ovaries revealed that ALA pretreatment significantly increased proline, soluble protein, and soluble sugar contents by 74.4%, 4.6%, and 36.19%, respectively, compared to untreated controls. Malondialdehyde (MDA) and hydrogen peroxide (H_2_O_2_) levels were significantly reduced in ALA-pretreated ovaries, whereas superoxide anion (O_2_^•−^) accumulation was unaffected. Both low-temperature (LT) and LT + ALA treatments significantly elevated O_2_^•−^ levels, indicating that ALA did not alleviate cold-induced O_2_^•−^ accumulation. Furthermore, ALA pretreatment significantly enhanced catalase (CAT), superoxide dismutase (SOD), and peroxidase (POD) activity levels by 9.14%, 47.51%, and 246.73%, respectively (Figure S1B).

### 2.2. Transcription Factor PpDof9 Interacted with the PpC3H37 Promoter

The previous research results showed that ALA promoted the expression of *PpWRKY18* by positively regulating *PpC3H37* and improved the cold tolerance of peach blossoms [26]. However, the mechanism by which ALA regulates the expression of *PpC3H37* is not clear. To explore the mechanism by which ALA regulates the expression of *PpC3H37*, we used ALA-treated peach blossoms under low-temperature conditions as experimental materials to construct a cDNA yeast library. Using the *PpC3H37* promoter as the bait (pHIS2-*proPpC3H37*), yeast cDNA library screening was conducted, and PpDof9 candidate proteins that might interact with the *PpC3H37* promoter were obtained.

**Figure 1:**
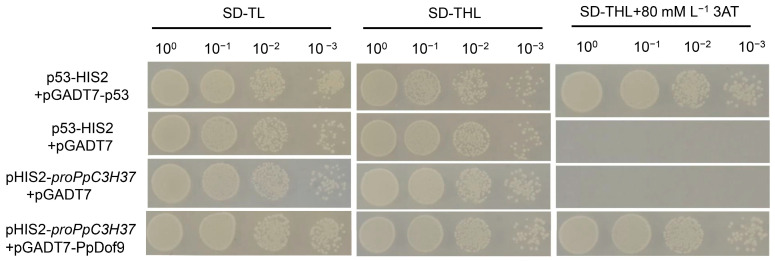
Yeast one-hybrid assay showing that PpDof9 interacted with the promoter of the *PpC3H37* in yeast. SD-TL: -trp, -leu; SD-TLH: -trp, -leu, -his; 3-AT: 3-amino-1,2,4-triazole.

The results of the yeast single-hybridization experiment indicated that PpDof9 interacted with the promoter of *PpC3H37* (Figure 1). The p53-HIS2 + pGADT7-p53 was used as a positive control and could grow normally on the SD/-trp/-leu/-his medium containing 80 mM 3-amino-1,2,4-triazole. Conversely, the negative control pHIS2-promoter of *PpC3H37* plus pGADT7 and p53-HIS2 plus pGADT7 proved that the operation of this experiment was correct. The pGADT7-PpDof recombinant plasmid was co-transformed into yeast with the *PpC3H37* promoter, namely pHIS2-promoter of *PpC3H37*. The results showed that they could grow normally on SD/-trp/-leu/-his containing 80 mM 3AT, indicating that *PpDof9* could activate the expression of the reporter gene, enabling pHIS2-promoter of *PpC3H37* to grow normally on the three-deficient plate containing 3AT.

### 2.3. Identification of PpDof Gene Family Members

**Figure 2:**
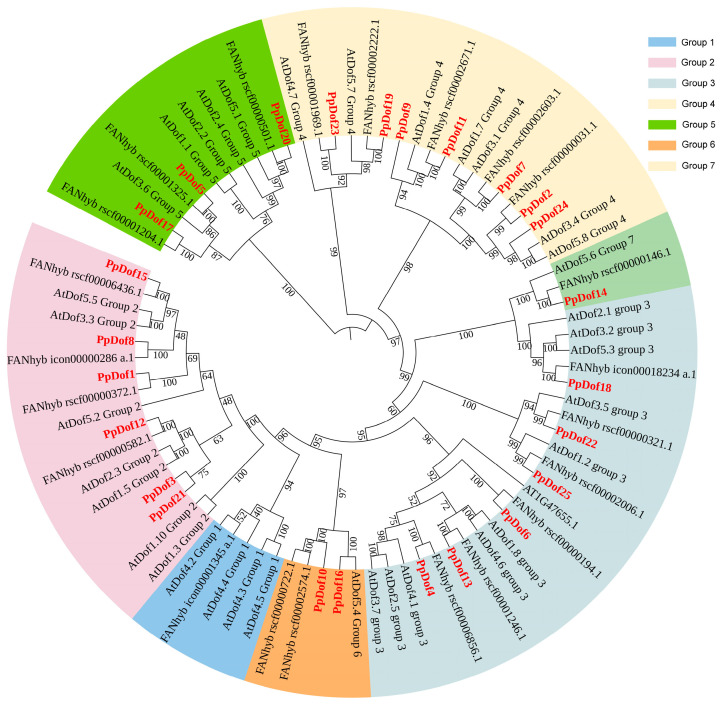
Phylogenetic trees of *Dof* genes in *Arabidopsis thaliana*, the strawberry, and the peach. Different colors represent different subfamilies.

In order to further elucidate the quantity of PpDof TFs in peaches, we performed a comprehensive identification of the *PpDof* gene family members and conducted subsequent bioinformatic analyses. The results revealed that the peach genome harbors 25 *PpDof* genes. To gain deeper insights into the evolutionary relationships of the peach Dof gene family, this study integrated peach Dof protein sequences with previously obtained *Arabidopsis thaliana* and strawberry Dof protein sequences. Using IQ-TREE (2.1.4-beta) software, we constructed a maximum likelihood (ML) tree, with the results shown in Figure 2. Following the subfamily classification criteria established for *Arabidopsis thaliana Dof* genes, the 25 peach *Dof* genes were categorized into subfamilies. The analysis revealed that these genes could be classified into six subfamilies (Groups 2–7). Notably, no corresponding peach *Dof* members were found in group 1 of *Arabidopsis thaliana*, where only a single strawberry *Dof* gene was present. This suggests that the peach may have undergone the loss of group 1 gene members during its evolutionary history. Regarding the distribution of peach *Dof* genes across subfamilies, group 2 and group 3 each contained six peach *Dof* members, group 4 contained seven, group 5 contained three, group 6 contained two, and group 7 had only one. The distribution pattern of members across subfamilies in the strawberry (also belonging to *Rosaceae*) was largely consistent with that of the peach. With the exception of group 1, each strawberry *Dof* gene clustered closely with its corresponding peach *Dof* counterpart in the phylogenetic tree, indicating a closer evolutionary relationship between peach and strawberry *Dof* genes.

**Figure 3:**
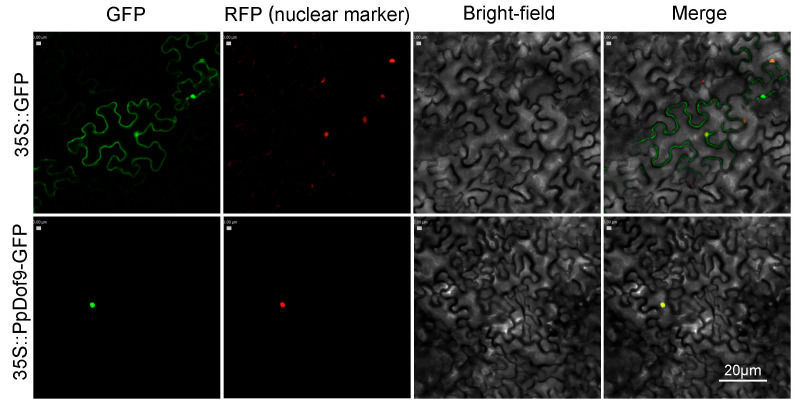
Subcellular localization of *PpDof9*. GFP: green fluorescent protein. RFP (nuclear marker): red fluorescent protein. Scale bar: 20 μm.

For convenient reference, these genes were systematically designated as *PpDof1* to *PpDof25*, reflecting the abbreviations for *Prunus persica* and their sequential order from top to bottom on their respective chromosomes. The physicochemical properties of the 25 PpDof proteins are summarized in Table 1. Our analysis of Table 1 shows that the PpDof proteins range in length from 162 to 515 amino acid residues. PpDof12 encodes the shortest protein (162 aa), while PpDof1 encodes the longest (515 aa). The relative molecular masses of the proteins span from 18,190.76 to 55,136.67 Da, exhibiting a positive correlation with their respective amino acid lengths. The predicted isoelectric points (pI) of the 25 PpDof proteins differed significantly. Nine proteins (PpDof1, PpDof2, PpDof8, PpDof14, PpDof15, PpDof16, PpDof18, PpDof22, PpDof25) have pI values below 7.0, indicating they are predicted to be acidic proteins. The remaining proteins are predicted to be basic. Among all members, PpDof25 exhibited the lowest pI (4.67), while PpDof20 had the highest pI (9.36). Subcellular localization predictions indicated that *PpDof3* is localized to the cytoplasm, *PpDof12* to the chloroplast, and all other family members are predicted to reside in the nucleus. It is expected that *PpDof9* is clearly localized in the nucleus of the guard cells of tobacco leaves (Figure 3). The instability indices of the PpDof proteins ranged from 35.43 to 65.06. All members except *PpDof3* and *PpDof24* exhibited instability indices greater than 40, suggesting that the majority of the PpDof proteins are unstable. The grand average of hydropathy (GRAVY) values for all PpDof proteins were negative, confirming that all are predicted to be hydrophilic proteins.

### 2.4. Chromosomal Localization and Synteny Analysis of the PpDof Gene Family in Peaches

**Figure 4:**
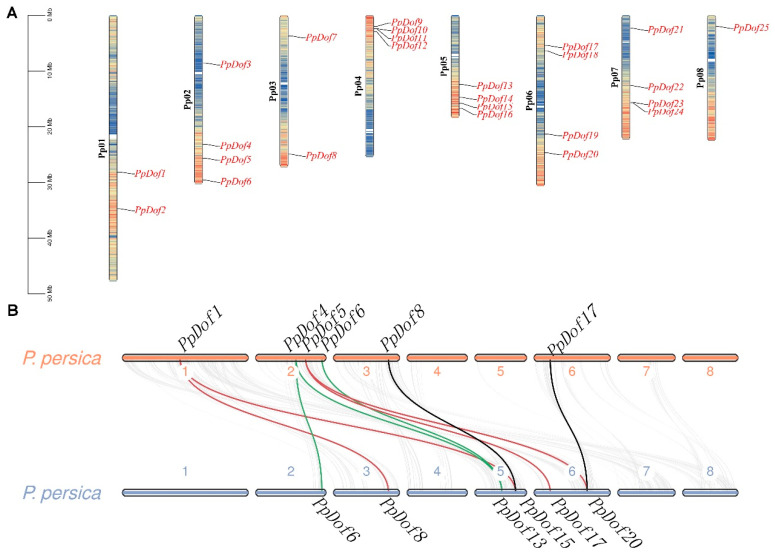
*PpDof* chromosome localization (**A**) and a collinearity analysis (**B**).

To gain a clearer understanding of the distribution of *PpDof* genes across the eight chromosomes of the peach and facilitate chromosome-level studies, a chromosomal localization analysis of the *PpDof* genes was performed using TBtools based on gene annotation information. The results are presented in Figure 4A. The *PpDof* genes exhibited an irregular distribution pattern across all chromosomes. Specifically, chromosomes 2, 4, 5, 6, and 7 each harbored four *PpDof* members. Chromosome 8 contained the fewest members, with only one family member (*PpDof25*). Chromosomes 1 and 3 each contained two members.

Interspecies synteny analyses, which compare the positions, gene structures, and sequences of homologous genes across multiple species, help determine evolutionary relationships. Genes located within a 200 kb genomic region on the same chromosome and sharing greater than 70% sequence similarity are generally considered to have arisen from tandem duplication events, a major mechanism for gene family expansion. Synteny and gene duplication events within the *PpDof* gene family were analyzed using MCScanX. As shown in Figure 4B, nine syntenic gene pairs were identified within the *PpDof* family: (*PpDof1*/*PpDof8*, *PpDof1*/*PpDof15*; *PpDof4*/*PpDof6*, *PpDof4*/*PpDof13*, *PpDof5*/*PpDof17*, *PpDof5*/*PpDof20*, *PpDof6*/*PpDof13*, *PpDof8*/*PpDof15*, *PpDof17*/*PpDof20*). This synteny likely resulted from large-scale chromosomal segmental duplications. A further analysis revealed four and three tandem duplication gene pairs on chromosomes 5 and 6, respectively (Figure 4). Collectively, these results indicate that both chromosomal duplication and gene duplication events likely played significant roles in the evolution of the Dof gene family In the peach.

### 2.5. Analysis of the Conserved Motifs of the PpDof Gene Family

**Figure 5:**
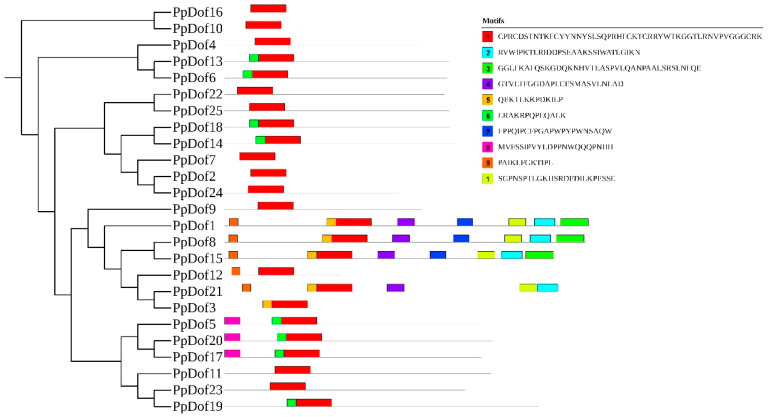
The motifs of the *PpDof* gene family.

Within the phylogenetic relationships of various families, most closely related genes consist of similar conserved motifs, indicating functional similarity among the *PpDof* family. As shown in Figure 5, motif 1 is present in all proteins and represents a highly conserved motif among *PpDof* genes.

### 2.6. Analysis of Cis-Regulatory Elements in the Promoters of PpDof Family Members

**Figure 6:**
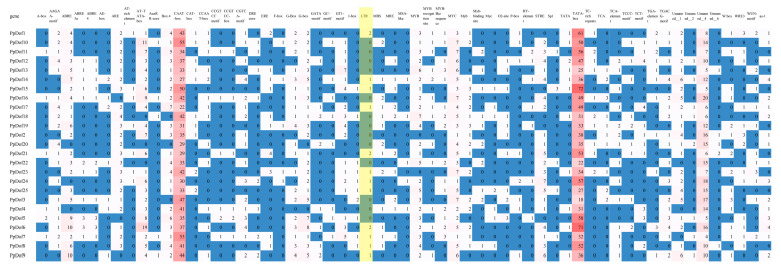
An analysis of cis-acting elements of *PpDof* gene family promoter.

As shown in Figure 6, the promoter regions of members in this gene family contain numerous cis-acting elements. These elements bind other proteins and activate various pathways. Beyond common light-responsive elements, cis-acting elements associated with plant hormones and abiotic stresses were also identified. The stress-related cis-elements detected include ABRE (abscisic acid-responsive element), ARE (anaerobic induction-responsive element), CAT-box (meristem expression), G-box (light-responsive element), RY-element (seed-specific regulatory element), TGA-box (auxin-responsive element; standard function may differ), MBS (drought-inducible element), and LTR (low-temperature responsive element). Notably, LTR elements were identified in *PpDof1*, *PpDof4*, *PpDof5*, *PpDof17*, *PpDof21*, *PpDof23*, *PpDof24*, *PpDof25*, *PpDof6*, *PpDof7*, *PpDof8*, and *PpDof9* (Figure 6).

### 2.7. ALA Regulated PpDof-Related Genes Under Cold Stress

**Figure 7:**
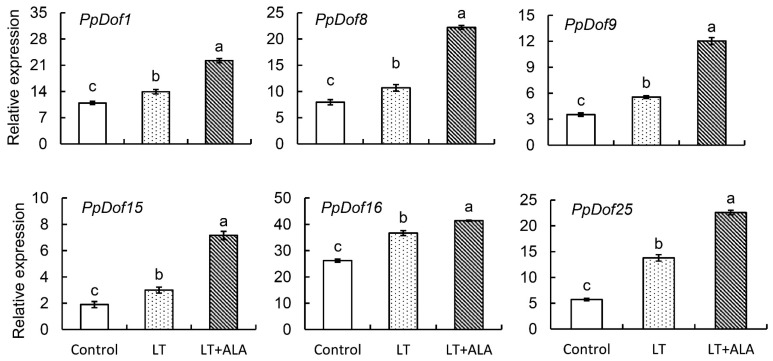
The highly expressed *PpDofs* genes in response to low-temperature stress and regulated by ALA. The treatments of flowers were defined as follows. ① Control: Cultured in distilled water + maintained at ambient temperature (22 °C/15 °C). ② Low-temperature treatment (LT): Cultured in distilled water + subjected to −3 °C for 6 h. ③ ALA treatment (ALA + LT): Pretreated with 50 mg·L^−1^ ALA + subjected to −3 °C for 6 h. The values are the means ± SE from three biological replicates. The same letters indicate no significant differences at *p* = 0.05 level.

To investigate the expression patterns of the *PpDofs* gene family members in peach flowers under low-temperature stress and following ALA pretreatment, we performed a qRT-PCR analysis. The results revealed distinct expression responses. Six genes (*PpDof1*, *PpDof8*, *PpDof9*, *PpDof15*, *PpDof16*, and *PpDof25*) were significantly upregulated under low-temperature stress, and this upregulation was further enhanced by ALA pretreatment (Figure 7). Seven genes (*PpDof3*, *PpDof10*, *PpDof11*, *PpDof17*, *PpDof20*, *PpDof21*, and *PpDof22*) exhibited no significant changes in expression in response to either low-temperature stress or ALA pretreatment compared with control groups. Four genes (*PpDof5*, *PpDof14*, *PpDof23*, and *PpDof24*) were significantly downregulated by low-temperature stress, and their expression levels were unaffected by ALA pretreatment. The *PpDof6* expression was responsive to low-temperature stress (significantly altered) but remained unaffected by ALA pretreatment. The *PpDof7* expression was unaltered by low-temperature stress but showed a significant response to ALA pretreatment (Figure S2).

## 3. Discussion

In actual agricultural production, late-spring cold spells and frosts (including phenomena such as ‘spring frost damage’) expose most fruit trees to low-temperature stress. This consequently results in significant yield losses [27,28]. As an environmentally friendly plant growth regulator, ALA enhances cold tolerance in fruit trees [29]. Yuan et al. (2024) [26] revealed a molecular mechanism mediated by the ALA-regulated PpC3H37-PpWRKY18 module in the cold stress response of peach pistils. ALA pretreatment enhances *PpC3H37* expression, thereby activating PpWRKY18 transcription. PpWRKY18 interacts with PpCBF1, participating in the regulation of cold tolerance via the CBF-dependent pathway. Crucially, PpCBF1 regulates the expression of *PpP5CS1* and *PpCOR1*, thereby enhancing osmoprotectant accumulation and improving low-temperature tolerance. Furthermore, PpWRKY18 directly targets the *PpPOD41* promoter region, positively regulating *PpPOD41* expression. This upregulation enhances POD activity, reduces reactive oxygen species (ROS) accumulation, and plays a positive role in mitigating freeze injury in peach flowers [26]. In this study, the *PpC3H37* promoter was used as bait in a yeast one-hybrid screen, which identified PpDof9 as a candidate transcription factor (Figure 1).

The Dof family represents a plant-specific class of TFs within the zinc finger superfamily [11]. These factors play crucial roles in plant growth, development, and stress responses [30,31]. While genome-wide identification of *Dof* families has been completed in model plants such as *Arabidopsis* [32], rice [33], and maize [34], the PpDof family remained uncharacterized. In this study, we identified 25 Dof TFs (designated PpDofs) within the peach genome (Figure 2, Table 1). Gene families often possess characteristic motifs. A sequence analysis revealed that all PpDof proteins share a conserved motif1 (Figure 5), suggesting its fundamental functional importance. An analysis of PpDof protein physicochemical properties indicated variations among individual members. Subcellular localization predictions showed that all *PpDof* members, except *PpDof3* and *PpDof12*, are localized to the nucleus. Subcellular localization experiments in tobacco revealed that *PpDof9* was localized to the nucleus, consistent with predictions (Figure 3). This nuclear localization aligns with their function as TFs regulating downstream gene expression [35]. Gene duplication events, a key driver of evolutionary diversification in plants [36,37], contribute to the expansion of the *PpDof* family. The expansion of the *PpDof* family appears driven by both segmental duplication (evidenced by 9 syntenic pairs) and tandem duplication events (noted on chromosomes 5 and 6), common mechanisms for the diversification of transcription factor families (Figure 4).

**Figure 8:**
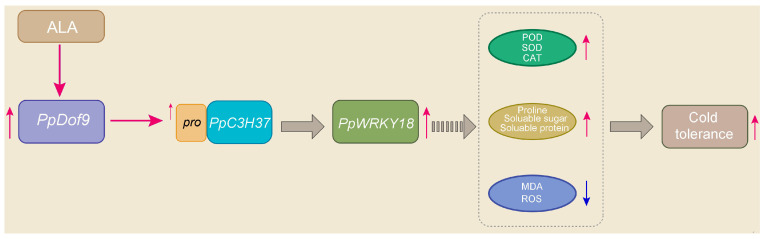
The possible regulatory pathway of *PpDof9* involvement in ALA-enhanced cold tolerance in peach flowers. Note: → represents a positive effect. The red arrow represents positive regulation and the blue arrow represents negative regulation.

Promoters, DNA sequences recognized by RNA polymerase for transcription initiation, are typically located several hundred to several thousand base pairs upstream of the transcription start site. The distribution of cis-acting elements within promoter sequences provides insights into potential gene function [38]. An analysis identified LTR retrotransposon elements in the promoters of *PpDof1*, *PpDof4*, *PpDof5*, *PpDof6*, *PpDof7*, *PpDof8*, *PpDof9*, *PpDof17*, *PpDof21*, *PpDof23*, *PpDof24*, and *PpDof25* (Figure 6), suggesting potential roles for these genes in peach cold tolerance. The qRT-PCR analysis showed that six genes (*PpDof1*, *PpDof8*, *PpDof9*, *PpDof15*, *PpDof16*, and *PpDof25*) were significantly upregulated under low-temperature stress, and this upregulation was further enhanced by ALA pretreatment (Figure 7). These findings establish the *PpDof* family as significant players in the transcriptional network governing cold stress adaptation in the peach, modulated by ALA, and provide crucial candidate genes (*PpDof1*, *PpDof9*, *PpDof8*, *PpDof25*) for future functional validation aimed at enhancing floral frost tolerance. Finally, we have summarized a possible regulatory pathway (Figure 8), whereby ALA enhances cold tolerance in peach blossoms by upregulating PpDof9 expression, which activates the PpC3H37 promoter, thereby enhancing PpWRKY18 expression. This molecular regulation leads to increased activities of the antioxidant enzymes SOD, POD, and CAT, along with elevated proline, soluble sugar, and soluble protein contents. Concurrently, it reduces MDA and ROS levels.

## 4. Materials and Methods

### 4.1. Plant Materials and Chemical Treatments

This study utilized six-year-old ‘Zijinhong 1’ nectarine trees cultivated at the National Peach and Strawberry Germplasm Repository of the Jiangsu Academy of Agricultural Sciences. Sampling and low-temperature treatment protocols followed Yuan et al. (2024) [26]. Flower branches approximately 50 cm long, exhibiting uniform bud developmental stages, were collected from the outer canopy of trees with similar growth vigor during the small bud stage. Apical and basal buds were removed, retaining a central section of approximately 20–30 cm in length bearing 10–15 pairs of flower buds per branch. Branches were randomly divided into three groups. Two groups were cultured in distilled water, while the third group was cultured in a solution containing 50 mg·L^−1^ ALA. All branches were maintained in an illuminated incubator (Ningbo, China, Ningbo Jiangnan instrument factory, GXZ-1000) under a 12/12 h (light/dark) photoperiod with a light intensity of 2000 lx during the light phase, a temperature regime of 22 °C/15 °C (day/night), and constant relative air humidity of 55%. The culture solution was renewed every three days. After one week of cultivation, when the majority of peach flowers had opened, the three groups of flower branches were assigned to the following three treatments, each with three biological replicates. Each replicate consisted of 30 flower branches, with each branch bearing 20–30 flowers. The treatments were defined as follows: ① Control (CK): Cultured in distilled water + maintained at ambient temperature (22 °C/15 °C). ② Low-temperature treatment (LT): Cultured in distilled water + subjected to −3 °C for 6 h. ③ ALA treatment (ALA + LT): Pretreated with 50 mg·L^−1^ ALA + subjected to −3 °C for 6 h. Following the treatments, all samples (including the control group) were immediately frozen in liquid nitrogen and stored at −80 °C for a subsequent analysis.

### 4.2. Physiological and Biochemical Parameter Determination in Peach Ovaries

Physiological and biochemical parameters of peach ovaries were determined as described by Yuan et al. (2024) [26].

### 4.3. Yeast One-Hybrid Assay

Total RNA extracted from processed ovary tissue was submitted to Shanghai OE Biotech Co., Ltd. for yeast library construction. The promoter region of *PpC3H37* (2000 bp upstream of the CDS) was cloned into the pHIS2 vector using the *EcoRI* and *SacI* restriction sites to generate the bait construct. Auto-activation assays demonstrated that 80 mM 3-amino-1,2,4-triazole (3-AT) was sufficient to suppress auto-activation by *proPpC3H37*. Yeast one-hybrid library screening was performed strictly according to the CLONTECH Matchmaker Gold Yeast One-Hybrid System manufacturer’s protocol [17]. Following sequencing and alignment of positive yeast clones, the candidate transcription factor PpDof9, potentially interacting with the *PpC3H37* promoter, was identified. The *PpDof9* coding sequence was subsequently cloned into the pGADT7 vector using the *EcoRI* and *BamHI* restriction sites to generate the prey construct. For one-to-one interaction validation, both the bait and prey constructs were co-transformed into Y187 yeast competent cells (Weidi Biotechnology, Shanghai, China; Cat# YC1020). All primers used in this study are listed in Supplementary Table S1.

### 4.4. Identification of Dof Genes in Peaches, Construction of a Phylogenetic Tree and Collinearity Analysis

The peach genome assembly and annotation files were retrieved from the Phytozome database [39]. Protein sequences were analyzed for the presence of the Dof domain (PF02701) using HMMER3.0 software [40] with an E-value cutoff of 1 × 10^−5^. Genes encoding proteins with a predicted *Dof* domain were further validated for domain integrity using the Conserved Domain Database (CDD) [41], applying an E-value threshold of 0.01. This process resulted in the identification of 25 candidate *Dof* family genes.

Protein sequences of *Arabidopsis thaliana* Dof TFs were retrieved from the TAIR database (https://www.arabidopsis.org/). Protein sequences of *Fragaria* × *ananassa* (strawberry) Dof TFs were obtained from the PlantTFDB database [42]. The protein sequences from each species were used for phylogenetic reconstruction. Multiple sequence alignment was first performed using MAFFT (v7.520) [43] with default parameters. Subsequently, a maximum likelihood (ML) tree was inferred using IQ-TREE (v2.4.0) [44]. The ‘-MFP’ parameter was employed to automatically select the best-fit substitution model, and the ‘-B 1000’ parameter was used to perform 1000 replicates of an ultrafast bootstrap analysis to assess node support values. The resulting tree was visualized and annotated using iTOL (v6) [45]. Subfamilies were delineated based on the established classification for *Arabidopsis thaliana* [46]. The Physcomitrium patens *PpDof* species tree was constructed following the same procedure.

We performed a collinearity analysis using JCVI (Python version) software [47].

### 4.5. Calculation of Basic Information for Gene Family Members

The chromosomal locations of these genes were visualized using TBtools (2024.1.11) software [17]. Gene IDs were subsequently renamed based on their sequential order along the chromosomes. Physicochemical properties of the proteins were calculated using TBtools, and the subcellular localization of the genes was predicted using the WoLF PSORT webserver (https://wolfpsort.hgc.jp/ accessed on 25 June 2020).

### 4.6. Subcellular Localization of PpDof9

The *PpDof9* coding sequence, excluding its termination codon, was amplified by PCR and cloned into the *BglII* and *SpeI* restriction sites of the pCAMBIA1302 vector (abbreviated as p35S) containing a C-terminal GFP tag. This was achieved using homologous recombination, generating the recombinant plasmid pCAMBIA1302- *PpDof9*-GFP. The resulting construct was subsequently transformed into *Agrobacterium tumefaciens* strain *GV3101*, and positive transformants were selected to obtain the agrobacterial culture.

Four-week-old *Nicotiana benthamiana* plants exhibiting uniform growth vigor were selected as the host for transient expression assays. Leaves were infiltrated with the *Agrobacterium* suspension (OD_600_ = 0.5–0.6). An *Agrobacterium* strain carrying the empty pCAMBIA1302-GFP vector (p35S-GFP) served as the control. The vector pCAMBIA2300-35S-*H2B*-mCherry [48] was used as a nuclear marker and was simultaneously injected into tobacco leaves in a volume ratio of 1:1 with *Agrobacterium* containing the target gene *PpDof*. The subcellular localization of the GFP fusion protein and nuclear marker was examined using a super-resolution laser scanning confocal microscope (LSM 800, Zeiss, Jena, Germany). To ensure comparability across samples, identical instrumental settings (including the laser intensity, detector pinhole diameter, and photomultiplier tube gain) were maintained during image acquisition. The primer sequences used in this study are listed in Table S1.

### 4.7. RNA Extraction, Reverse Transcription, and RT-qPCR Analysis

Total RNA was extracted using the MolPure^®^ Plant RNA Kit (Yeasen Biotechnology (Shanghai, China) Co., Ltd., Cat# 19291ES50). Reverse transcription was performed using the Hifair^®^ II 1st Strand cDNA Synthesis Kit (gDNA digester plus) (Yeasen Biotechnology (Shanghai, China) Co., Ltd., Cat# 11121ES60) according to the manufacturer’s instructions.

Quantitative reverse transcription PCR (RT-qPCR) was carried out on an ABI 7500 Real-Time PCR System (Applied Biosystems, Foster City, CA, USA). The thermal cycling protocol consisted of an initial denaturation step at 95 °C for 5 min, followed by 40 cycles of denaturation at 95 °C for 10 s, and combined annealing–extension at 60 °C for 30 s. A dissociation curve analysis step was included at the end of the run. The *PpTEF2* was used as the reference gene for normalization. Primer sequences for the target genes are listed in Supplementary Table S1. Each sample was assayed in three biological replicates. Relative gene expression levels were calculated using the 2^−ΔΔCt^ method [49].

### 4.8. Data Analysis

Experiments were performed with at least three independent biological replicates per group, with random sampling. Data were organized and visualized using Microsoft Excel 2019. Statistical significance was assessed via a one-way or two-way analysis of variance (ANOVA) followed by Duncan’s multiple range test, with significance set at *p* < 0.05. Figures were processed using Adobe Photoshop 2017.

## 5. Conclusions

This study elucidates the regulatory mechanism whereby ALA enhances peach cold tolerance via the PpC3H37-PpWRKY18 module. We identified *PpDof9* as a key transcription factor directly binding the *PpC3H37* promoter. Characterization of the peach *Dof* family (25 genes) revealed expansion via segmental and tandem duplications. Crucially, ALA pretreatment selectively upregulates specific cold-responsive *PpDofs*, including *PpDof1*, *PpDof8*, *PpDof9*, and *PpDof25*, under low-temperature stress. These findings demonstrate ALA’s role in modulating *PpDof* TF expression and identify prime candidates for improving floral frost tolerance.

## Figures and Tables

**Table 1:** Detailed information of *PpDof* gene families in *Prunus persica*.

Sequence ID	Number of Amino Acid	Molecular Weight	Theoretical pI	Instability Index	Aliphatic Index	Grand Average of Hydropathicity	Subcellular Localization Prediction
*PpDof6*	314	34,776.11	7.76	49.88	45.35	−0.904	nucl
*PpDof5*	359	38,910.01	8.74	57.24	51.89	−0.736	nucl
*PpDof3*	202	22,168.98	7.61	35.43	65.89	−0.444	cyto
*PpDof4*	278	30,370.41	8.15	40.33	51.69	−0.799	nucl
*PpDof25*	317	35,045.02	4.67	51.54	57.22	−0.704	nucl
*PpDof18*	318	35,194.72	6.78	58.19	49.4	−0.845	nucl
*PpDof19*	443	46,689.04	8.72	56.54	48.96	−0.763	nucl
*PpDof17*	362	39,227.18	8.77	51.13	57.13	−0.788	nucl
*PpDof20*	378	39,718.75	9.36	57.83	46.24	−0.665	nucl
*PpDof12*	162	18,190.76	9.32	51.75	52.35	−0.688	chlo
*PpDof9*	278	30,732	8.76	65.06	55.83	−0.721	nucl
*PpDof10*	292	31,543.29	9.01	64.41	44.18	−0.942	nucl
*PpDof11*	376	40,988.84	8.09	54.3	49.34	−0.824	nucl
*PpDof15*	466	50,778.37	5.35	61.72	54.68	−0.855	nucl
*PpDof13*	317	34,615.2	9	50.73	56.94	−0.773	nucl
*PpDof16*	326	34,843.02	6.59	61.69	50.03	−0.706	nucl
*PpDof14*	327	36,439.52	6.55	41.01	47.71	−0.815	nucl
*PpDof23*	339	36,002.86	8.98	58.97	56.43	−0.537	nucl
*PpDof24*	245	25,122.85	8.51	35.98	47.43	−0.448	nucl
*PpDof22*	310	33,657.01	6.26	44.96	57.29	−0.653	nucl
*PpDof21*	474	51,461.92	7.71	55.94	66.01	−0.524	nucl
*PpDof8*	509	55,278.07	6.05	55.43	51.38	−0.871	nucl
*PpDof7*	226	24,245.53	8.74	51.36	45.75	−0.857	nucl
*PpDof2*	283	30,140.23	5.67	46.64	44.42	−0.575	nucl
*PpDof1*	515	55,136.67	5.88	52.7	54.25	−0.707	nucl

## Data Availability

The data underlying this article are available in this paper. The datasets in this article were derived from sources in the public domain, including the National Center for Biotechnology Information (National Center for Biotechnology Information (nih.gov)) and the *Arabidopsis* Information Resource (https://www.arabidopsis.org).

## Supplementary Materials

The following supporting information can be downloaded at https://www.mdpi.com/article/10.3390/ijms26157509/s1.
