# Supplementary material for: Systematic Analysis of Dof Gene Family in Prunus persica Unveils Candidate Regulators for Enhancing Cold Tolerance
Source: Int J Mol Sci. 2025 Aug 4;26(15):7509. doi: 10.3390/ijms26157509 (PMC12347117; doi:10.3390/ijms26157509)
Supplement: Supplementary file 1 [file ijms-26-07509-s001.zip › Figure S1.pdf]

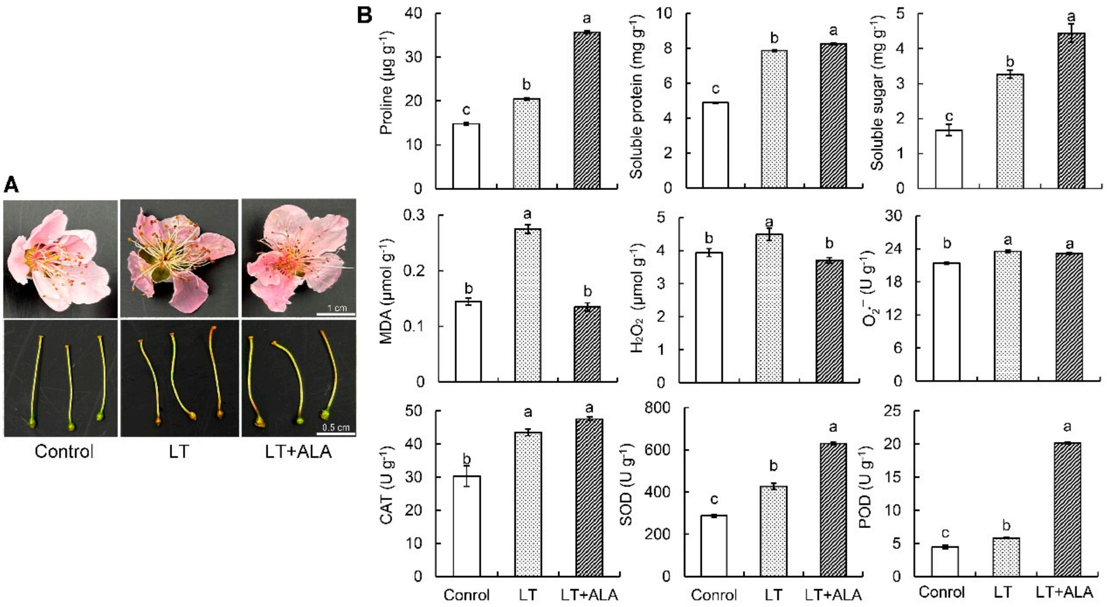

Fig.S1 The effects of ALA on the flower organs and physiological and biochemical indicators of peach blossoms under low-temperature treatment

A: The effect of ALA on the phenotype of peach blossom organs under low-temperature treatment; B: The influence of ALA on the physiological and biochemical indicators of peach gynoecium under low-temperature treatment. Different lowercase letters indicate significant differences at a significance level of $p = 0.05$. Scale bar: 1 cm/0.5 cm.

Note: this part of the content has been submitted to Acta Horticulturae Sinica.
