# Supplementary figures and images for: Systematic Analysis of Dof Gene Family in Prunus persica Unveils Candidate Regulators for Enhancing Cold Tolerance
Source: Int J Mol Sci. 2025 Aug 4;26(15):7509. doi: 10.3390/ijms26157509 (PMC12347117; doi:10.3390/ijms26157509)

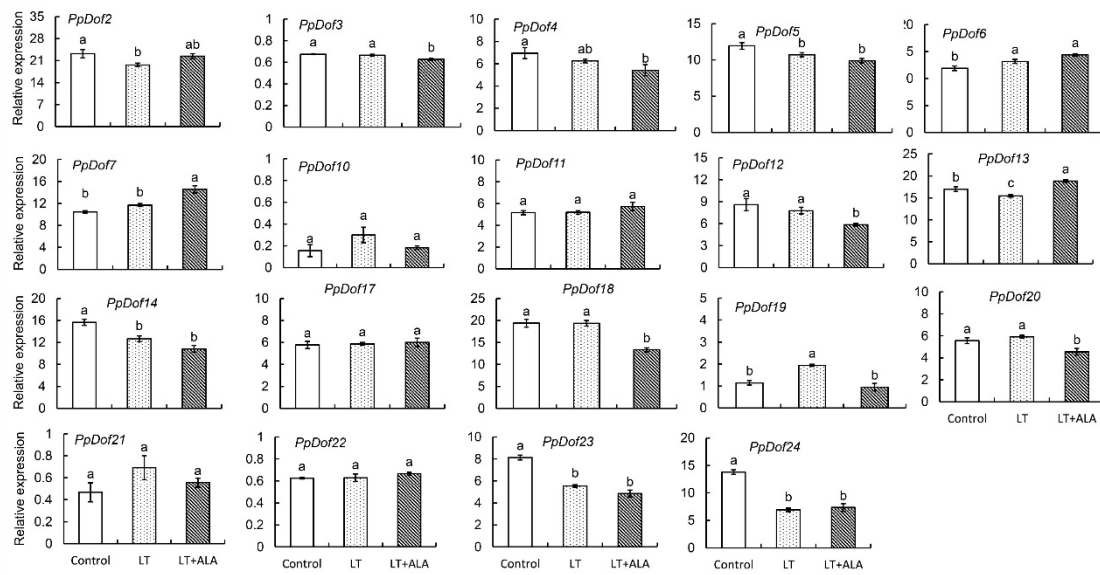

Figure S2 The expression patterns of other *PpDofs* genes under low-temperature stress.

Supplement: Supplementary file 1 [file ijms-26-07509-s001.zip › Figure S2.pdf]
